# Beta Distribution Network Modeling Improves Biological Integration of Multi-Omics Data

**DOI:** 10.1093/geroni/igaf122.2878

**Published:** 2025-12-31

**Authors:** Heeju Noh, Max Robinson, Lance Pflieger, Noa Rappaport

**Affiliations:** Institute for Systems Biology, Seattle, Washington, United States; Institute for Systems Biology, Seattle, Washington, United States; Phenome Health, Seattle, Washington, United States; Institute for Systems Biology, Buck Institute, Phenome Health, Seattle, Washington, United States

## Abstract

Correlation based clustering of multi-omics data typically results in platform-specific modules rather than biologically meaningful cross-platform associations, as technical variation and data structure differences between omics platforms dominate the correlation patterns.We introduce a novel network model that fits a Beta distribution to analyte-analyte correlations. First, correlations across different platforms are standardized by aligning Beta distribution shapes uniformly. Our approach then constructs an analyte relationship network by identifying outlier correlations against a null model background. We generated synthetic data which mimics realistic multi-omics profiles, where intra-omics correlations are generally stronger than inter-omics correlations. Next, we evaluate clustering performance using synthetic datasets, comparing our method to the standard Weighted Correlation Network Analysis (WCNA), which constructs networks based on a power-law distribution. Our method demonstrated higher clustering accuracy (p = 1.3e-13) and purity (p = 2.22e-16) compared to WCNA. The improvement was particularly notable at higher signal-to-noise ratios and fewer reference clusters. We further tested our method on human multi-omics cohort data (Arivale Wellness Study), our strategy outperformed the standard WCNA, achieving a higher average silhouette score and improved entropy, leading to better clustering of proteins, metabolites, and laboratory analytes. This approach is powerful for integrating multimodal molecular data in a biologically meaningful way, enabling the discovery of cross-platform relationships that reveal functional connections across biological layers

